# New adhesive traps to monitor urban mosquitoes with a case study to assess the efficacy of insecticide control strategies in temperate areas

**DOI:** 10.1186/s13071-015-0734-4

**Published:** 2015-02-28

**Authors:** Beniamino Caputo, Annamaria Ienco, Mattia Manica, Vincenzo Petrarca, Roberto Rosà, Alessandra della Torre

**Affiliations:** Dipartimento di Sanità Pubblica e Malattie Infettive, Università di Roma “Sapienza”, Piazzale Aldo Moro 5, 00185 Rome, Italy; Dipartimento di Biologia e Biotecnologie, Università di Roma “ Sapienza”, Piazzale Aldo Moro 5, 00185 Rome, Italy; Dipartimento di Biodiversità ed Ecologia Molecolare, Centro Ricerca e Innovazione, Fondazione Edmund Mach, San Michele all’Adige, TN Italia

**Keywords:** *Ae. albopictus*, Sticky trap, Vector control, Catch basins, Larvicide, Insecticide spraying

## Abstract

**Background:**

Urban mosquitoes in temperate regions may represent a high nuisance and are associated with the risk of arbovirus transmission. Common practices to reduce this burden, at least in Italian highly infested urban areas, imply calendar-based larvicide treatments of street catch basins – which represent the main non-removable urban breeding site – and/or insecticide ground spraying. The planning of these interventions, as well as the evaluation of their effectiveness, rarely benefit of adequate monitoring of the mosquito abundance and dynamics. We propose the use of adhesive traps to monitor *Aedes albopictus* and *Culex pipiens* adults and to evaluate the efficacy of insecticide-based control strategies.

**Methods:**

We designed two novel types of adhesive traps to collect adult mosquitoes visiting and/or emerging from catch basins. The Mosquito Emerging Trap (MET) was exploited to assess the efficacy of larvicide treatments. The Catch Basin Trap (CBT) was exploited together with the Sticky Trap (ST, commonly used to collect ovipositing/resting females) to monitor adults abundance in the campus of the University of Rome “Sapienza” - where catch basins were treated with Insect Growth Regulators (IGR) bi-monthly and Low-Volume insecticide spraying were carried out before sunset - and in a nearby control area.

**Results:**

Results obtained by MET showed that, although all monitored diflubenzuron-treated catch basins were repeatedly visited by *Ae. albopictus* and *Cx. pipiens*, adult emergence was inhibited in most basins. Results obtained by ST and CBT showed a significant lower adult abundance in the treated area than in the untreated one after the second adulticide spraying, which was carried out during the major phase of *Ae. albopictus* population expansion in Rome. Spatial heterogeneities in the effect of the treatments were also revealed.

**Conclusions:**

The results support the potential of the three adhesive traps tested in passively monitoring urban mosquito adult abundance and seasonal dynamics and in assessing the efficacy of control measures. ST showed higher specificity for *Ae. albopictus* and CBT for *Cx. pipiens.* The results also provide a preliminary indication on the effectiveness of common mosquito control strategies carried out against urban mosquito in European urban areas.

**Electronic supplementary material:**

The online version of this article (doi:10.1186/s13071-015-0734-4) contains supplementary material, which is available to authorized users.

## Background

In its native range, the mosquito species *Aedes albopictus* [*Stegomyia albopicta*] is present throughout much of the Oriental region from the tropics to northern China and North Korea. In recent decades, modern transportation has globalized the species most notably to much of the New World and several European countries [1,2]. Apart from Albania [3] where it was present since the mid-1970s (and possibly earlier), Italy was the first country in Europe (1990) with widespread infestation and where the densities in urban areas became a serious nuisance, especially due to the species aggressive day-time biting behaviour [4-8]. Moreover, Italy was the first European country to experience an outbreak of Chikungunya virus (CHIK) entirely sustained by *Ae. albopictu*s [9]. In fact, the species is a competent vector of several arboviruses [10-12] and has been responsible for major epidemics of CHIK in islands of Indian ocean and in India in 2005–2006 [13,14], as well as of the indigenous transmission of Dengue virus in Japan during the World War II [15,16] and, more recently, in the south of France [17,18] and Croatia [19]. These latest events highlighted the necessity to elaborate preparedness for response to autochthonous virus transmission in Europe [20]. In fact, due to the absence of commercially available vaccines against these arboviroses, vector control is the only effective measure presently available to stop an epidemic. In case of arboviral outbreaks in Europe, guidelines by the European Centre for Disease Prevention and Control, as well from the Italian National Health Institute (Istituto Superiore di Sanità, ISS), suggest integration of different strategies (e.g. public health education, larval breeding places reduction, biological control of non-removable potential larval sites such as catch basins, and insecticide treatments against adults mosquitoes) to reduce *Ae. albopictus* densities and control pathogen transmission [20,21]. A similar integrated approach, based on the use of adulticides restricted to specific areas (e.g. cemeteries, school yards and hospitals) or situations of high concentration of hosts (e.g. fairs, etc.), is also suggested by ISS to prevent the settlement of high *Ae. albopictus* densities. However, despite the ISS indications, adulticide treatments are largely implemented in Italian urban areas by both public and private companies with the sole aim to reduce the very high biting nuisance to the citizens. Main control measures usually involve treatments of catch-basins (considered as the main non-removable urban larval sites for *Ae. albopictus* and *Culex pipiens* [21-23]) with Insect-Growth-Regulators (IGR, which interfere with larval development and inhibit adult emergence) and spraying of pyrethroid and/or pyrethrum-based adulticides by truck-mounted cannon spray atomizers or portable thermal foggers.

Currently, the most widely used method to detect and monitor *Ae. albopictus* populations is the counting of eggs collected by ovitraps, which are small black containers resembling typical oviposition sites [24,25]. Ovitraps have been sometimes also used to carry out evaluations of the efficacy of treatments against *Ae. albopictus* in urban areas [26,27]. The advantages of ovitraps are that they are inexpensive and sensitive for the detection of *Ae. albopictus*, thus allowing large scale surveillance and monitoring schemes. However, ovitraps have also important constraints. First, there are theoretical controversies about the use of ovitrap data for assessing adult populations, particularly at high adult densities [28]. Second, in areas where species with similar egg/larval morphology are present (e.g. *Ae. albopictus* and *Ae. aegypti*), eggs and larvae need to be maintained until adult emergence for species identification [29]. These constraints are overcome by BG-Sentinel traps (BG-traps; Biogents, Regensburg, Germany), which have been specifically designed to actively collect host-seeking *Ae. albopictus* females (but also collect associated males, [30,31]) and are being increasingly used to determine the species abundance [31-33]. Recently, BG-traps have been also successfully exploited to evaluate the efficacy of integrated *Ae. albopictus* control activities in New Jersey, USA [27,34]. However, BG-traps have several limiting operational constraints (e.g. need of a power-supply, of CO_2_/lure release and of daily activation/maintenance, large size and high individual cost), which make their large scale exploitation very laborious and expensive. Finally, alternative to ovitraps and BG-traps, adhesive traps have been exploited to monitor ovipositing *Ae. albopictus* females attracted by a small water-container similar to an ovitrap. Adhesive traps overcome the limits of both ovitraps and BG-traps, but are less sensitive than ovitraps at very low densities [35] and collected adults, contrary to those collected by BG-traps, are difficult to be freed from glue in order to keep them for further analyses (e.g. molecular genetics analyses) and are not suitable for arbovirus search. Moreover, several designs of adhesive traps have been proposed [36-38] and so far used exclusively for research, and a standardized model is not yet accepted for routine monitoring activities.

The objective of the present study was to propose the use of adhesive traps not only to monitor urban mosquito abundance, but also to evaluate the efficacy of insecticide-based control strategies, such as those typically implemented in Italian urban areas infested by *Ae. albopictus* (and *Culex pipiens s.l.,* hereafter *Cx. pipiens*). To this aim we tested: i) a newly designed Mosquito Emerging Trap to assess adult emergence from catch basins treated with IGR-analogs, and ii) two adhesive trap designs to monitor adults abundance in relation to insecticide spraying in a study area in Rome: a) the Sticky Trap, already shown to provide estimates of species abundance correlated with those obtained by ovitraps [35] and exploited to study its behaviour in urban areas [39,40], and b) the Catch Basin Trap, *ad hoc* designed for large scale passive monitoring of urban mosquitoes associated to catch basins.

## Methods

### Trap design

**Figure 1:**
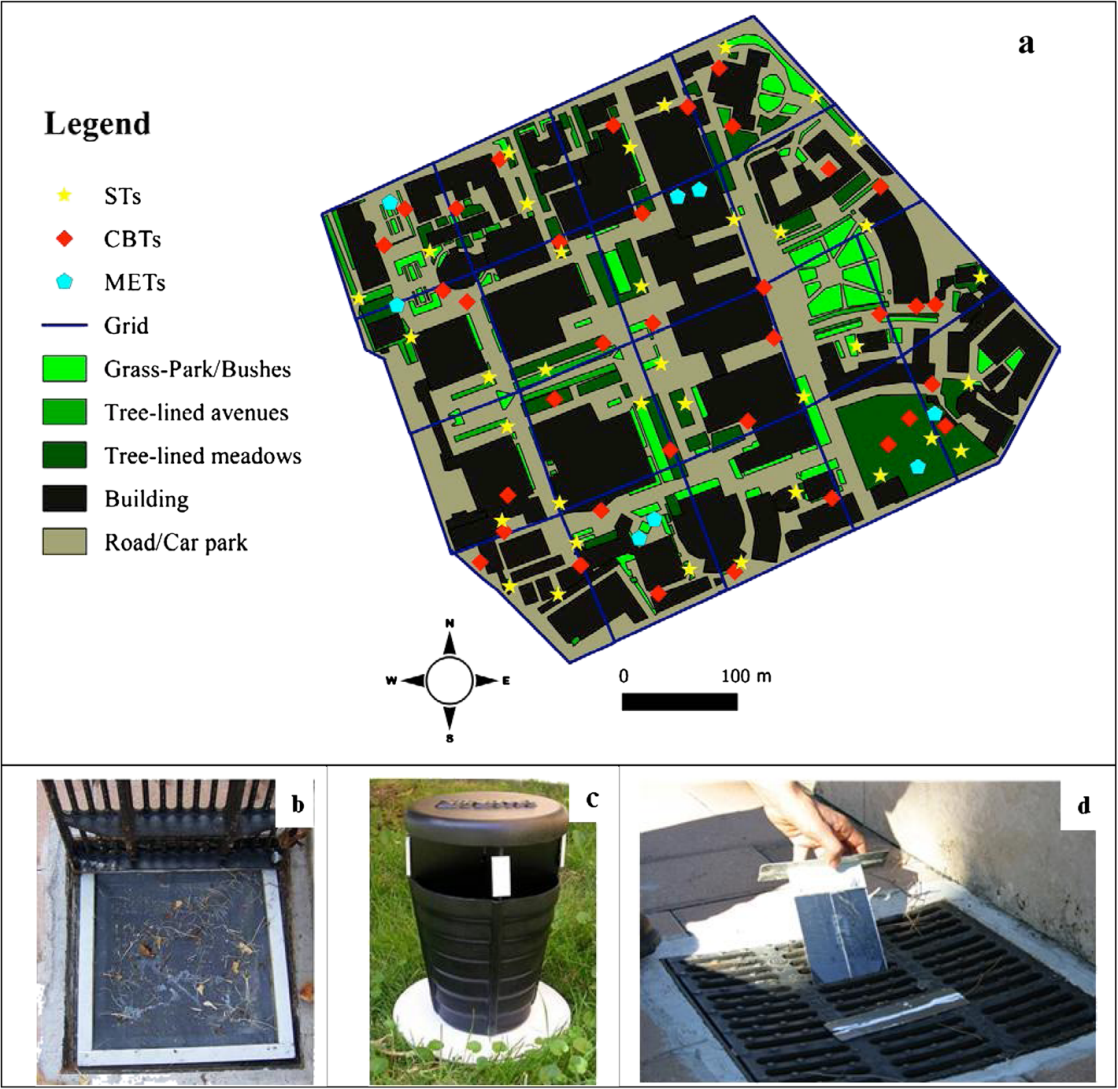
**Map of the campus of University of Rome “Sapienza”.** Sketch map (1:3800 scale, **a)** of the area treated with insecticides, including the operational subdivision into a 18-cell grid and the location of 8 Mosquito Emerging Traps (MET, **b)** 36 Sticky Traps (ST, **c)** and 36 Catch Basin Traps (CBT, **d)**. The map was obtained by manual digitizing via the software Quantum GIS (QGIS Development Team, 2013. QGIS Geographic Information System. Open Source Geospatial Foundation Project).).

Three types of adhesive traps have been used in the present study:

- the Mosquito Emergence Trap (MET; Figure 1b), consisting of a net panel coated with commercially marketed rat-glue (DeBello, Zapi Chemical Industries SpA) on both faces and fixed with ‘velcro’ to an aluminium-frame positioned under the drain-grid. MET of three sizes (40×40 cm, 44×44 cm, 55×55 cm) were used in relation to the sizes of the specific catch basin to be surveyed.
- the Sticky-Trap (ST, [35]; Figure 1c); the Catch Basin Trap (CBT; Figure 1d) consisting of a black panel (17×10 cm) set in an aluminium frame equipped with a top (18×5 cm) to maintain the frame perpendicular to the drain-grid. A transparent sheet manually coated with rat-glue at both sides is fixed at each side of the panel; the area of surface coated with glue was equal to that of the four adhesive panels in the ST.

### Insecticide treatments

Insecticide treatments against mosquito larvae and adults were carried out during summer 2012 on the campus of the University of Rome “Sapienza” (~22 hectares; hereafter “treated area”), which represents a highly urbanized area in Rome [39]. The campus is mostly characterized by buildings, roads, car parks and small green areas with trees and/or hedges and, at its eastern extremity, by a small botanical garden (Figure 1a).

Catch basins in the campus were treated with a larvicidal IGR (Flubex, 2 g tablets with 2% of diflubenzuron, I.N.D.I.A.. Industrie Chimiche SRL). One tablet was applied every second week (starting May 2012) to each of the 166 catch basins in the treated area, including those that were dry to avoid risk of production of larvae in case of rain.

A water-based 0.5% TERBUTIN formulation (ZEP ITALIA SRL .: 1.5 g pyrethrum 50% extract; 8 g permethrin, 2.64 g piperonyl butoxide for 100 g of product) was sprayed on July 26th and on August 23rd 2012. Cold Low-Volume (LV) spraying (droplet size < 50 μm) was carried out by a cannon spray atomizer (series “ELITE” Spray Team snc) mounted on a flatbed truck, with the boom angled at 45-70°. The vehicle was driven along all major roads in the campus (light-brown in Figure 1a) at an average speed of 15 km/h. Spraying was carried out 60–90 minutes before sunset and was concluded at dusk. All treatments were carried out by qualified technicians of a private company (SOGEA s.r.l.).

### Evaluation of insecticide treatments

The MET was used to assess the number of adults visiting and emerging from catch basins. Each week (from August 1th to September 28th), eight METs were positioned for 48 hours in two catch basins in each of the quadrants (North, N; East, E; South, S; West, W) of the treated area (Figure 1a) and in two basins in the untreated area (UN). This corresponds to the enclosed garden (the Institute of Anatomy, ~1 hectare at ~300 m from the treated area see [41] for details), where no treatments were performed. Before positioning the METs, mosquitoes possibly resting on the walls of the catch basin were chased away by a stick, to be sure that mosquitoes glued to MET’s inner face were freshly emerged ones. The adhesive panels were brought to the laboratory and glued mosquitoes were morphologically identified and counted under a stereo-microscope.

The CBT and the ST were used to assess the combined effect of larvicidal and adulticidal treatments. The treated area was sub-divided into a grid of eighteen 100×100m-cells and 2 STs and 2 CBTs were positioned in each cell, for a total of 72 traps (Figure 1a). Ten STs and ten CBTs were positioned in the untreated area. Sticky panels in ST and CBT were replaced on a weekly basis. Glued mosquitoes were brought to the laboratory and glued mosquitoes were morphologically identified and counted under a stereo-microscope [42]. Monitoring was carried out for 10 weeks (from July 12th to September 20th 2012). Monthly mean temperatures were 27.3°C, 30.2°C and 20.9°C in July, August and September, respectively. Rainfall was negligible in July and August, while nine days of rain were recorded in September.

To assess the impact of the adulticides and compare this to published data, an algebraic variation of Henderson’s method [43] was employed using the formula: Percentage control = [100 − (T/U) 100] where T is the post-application weekly mean of mosquito counts divided by the pre-application weekly mean in the treatment site, and U is the post-application weekly mean divided by the pre-application weekly mean in the untreated site.

### Statistical analysis

A generalized linear mixed model (GLMM) with negative binomial error term was carried out to compare the number of adult mosquitoes visiting the catch basins (i.e. glued in the outer side of METs) between treated and untreated sites. The response variables were the number of males, females and total *Ae. albopictus* and *Cx. pipiens*. Site of trapping (four sites for treated area, corresponding to the N, E, S and W quarters, and one for untreated area) was the explanatory variable and date of collection was included into models as random effect.

The impact of larvicides on the emergence of *Ae. albopictus* and *Cx. pipiens* was assessed by exact Fisher’s tests, comparing presence/absence of mosquitoes (glued to the inner side of METs) between treated and untreated sites. Differences were considered significant when α < 0.05 and all tests were two tailed. In this case, a Fisher’s test were preferred to a Generalized Linear Model (GLM) with binomial error as presence/absence of mosquitoes glued in the inner side of METs perfectly separates zeroes and ones among treated and untreated sites.

A GLM with negative binomial error term was carried out to investigate the relationship between ST- and CBT-counts in the overall sample, as well as in treated and in untreated sites. In this case, ST-counts were chosen as the response variable and CBT-counts as explanatory variable.

Four GLMMs with negative binomial error term were carried out to investigate the effect of treatments (treated vs. untreated site) and collection method (ST vs. CBT) on adult mosquito abundance. The response variables were mosquito counts for *Ae. albopictus* and *Cx. pipiens* separately considering specific models for male and female mosquitoes. Explanatory variables were treatments, collection method and their interaction. To account for possible temporal autocorrelation, date of collection was included into models as a random effect. In addition, a geostatistical variogram was applied to model residuals to evaluate if there was any spatial autocorrelation. In order to evaluate the temporal effect of adulticide treatments we carried out GLMMs using three different datasets corresponding to: i) the first two weeks of the trapping period that did not include adulticide treatments; (ii) the first six weeks of trapping that include the first adulticide treatment; (iii) the whole trapping period (ten weeks) that included both adulticide treatments. On the other hand, larvicidal-treatments were applied continuously throughout the trapping season, as explained in the previous section.

Four additional GLMMs with negative binomial error term were performed to assess the effect of spatial heterogeneity and collection method in the treated site. As above, the response variables were mosquito counts of the two species (*Ae. albopictus* and *Cx. pipiens*) and gender, while fixed effects were the sampling location (cell), trap types (ST and CBT) and their interaction. Date of collection was included as a random effect to account for temporal autocorrelation. In addition, a geostatistical variogram was applied to model residuals to evaluate if there was any spatial autocorrelation. In this case, the GLMMs were carried out only in the overall 10-week dataset.

Analysis were performed using R 3.0.3 [44] using the glmmADMB package [45,46].

## Results

The two novel adhesive traps designed to collect adult mosquito visiting and/or emerging from catch-basins (i.e. the MET, Figure 1b, and the CBT, Figure 1d) were both shown to collect females and males of the two mosquito species know to be associated with street catch basins in Italian urban areas, i.e. *Ae albopictus* and *Cx. pipiens* (Tables 1 and 2). Comparison of CBT vs ST performance in the untreated area showed that the ratio between *Ae. albopictus* and *Cx. pipiens* was slightly over 1 in CBT, and ca. 5 in ST (Table 2; Fisher’s Exact Test, p < 0.001).

**Table 1:** **Descriptive statistics of** ***Aedes albopictus*** **and** ***Culex pipiens*** **collected by Mosquito Emerging Trap**

	***Aedes albopictus***				***Culex pipiens***			
	**Females**		**Males**		**Females**		**Males**	
	**Outer face**	**Inner face**	**Outer face**	**Inner face**	**Outer face**	**Inner face**	**Outer face**	**Inner face**
**N**	1.9 (±0.3)	0	2.1 (±0.4)	0	1.3 (±0.3)	0	0.4 (±0.1)	0.1 (±0.1)
**E**	3.9 (±0.5)	1.6 (±0.3)	2.5 (±0.4)	2.5 (±0.4)	2.0 (±0.2)	1.4 (±0.3)	1.1 (±0.3)	2.2 (±0.4)
**S**	2.0 (±0.4)	0	1.6 (±0.4)	0	1.4 (±0.3)	0	0.7 (±0.2)	0.1 (±0.1)
**W**	3.4 (±0.4)	0	2.6 (±0.5)	0	1.5 (±0.3)	0	0.7 (±0.2)	0.1 (±0.1)
**UN**	5.8 (±0.6)	2.9 (±0.3),	4.1 (±0.6)	5.1 (±0.6)	3.4 (±0.5)	2.1 (±0.3)	2.1 (±0.4)	3.7 (±0.5)

Mean counts /trap/48 hours (±standard error) in Mosquito Emerging Trap in two Diflubenzuron-treated catch basins located in each of quarters (N, E, S, W) of the treated area and two catch basins in the untreated area (UN) during N = 15 samplings in 2012.

**Table 2:** **Descriptive statistics of** ***Aedes albopictus*** **and** ***Culex pipiens*** **collected by Sticky trap and Catch Basin Trap**

		***Aedes albopictus***		***Culex pipens***		***Aedes albopictus/Culex pipiens*** **ratio**	
**Site**	**Traps (N/area)**	**Females**	**Males**	**Females**	**Males**	**Females**	**Males**
**Treated**	ST (36)	4.5 (±0.2)	2.4 (±0.1)	1.6 (±0.1)	0.9 (±0.1)	2.7	2.6
	CBT (36)	1.8 (±0.1)	1.1 (±0.1)	1.2 (±0.1)	0.7 (±0.1)	1.5	1.6
**Untreated**	ST (10)	10.9 (±0.9)	6.1 (±0.5)	2.3 (±0.2)	1.1 (±0.1)	4.7	5.3
	CBT (10)	4.3 (±0.5)	3.0 (±0.5)	3.7 (±0.3)	2.1 (±0.2)	1.2	1.4

Mean counts/trap (±standard error) collected by 36 Sticky Traps (ST) and 36 Catch Basin Traps (CBT) in the treated area and in 10 STs and 10 CBTs in the untreated one during the overall 10-week sampling in 2012.

### Evaluation of the efficacy of IGR-treatments on mosquito emergence from street catch basins using the Mosquito Emerging Trap (MET)

Two Diflubenzuron-treated catch basins located at each of N, E, S and W quarters of the treated area and two catch basins in the untreated area (UN) were monitored using the MET. All METs were visited by mosquitoes (Table 1, “outer face” column). METs located in the E and W quarters collected a significant higher number of *Ae. albopictus* females than those in N and S ones, while no differences were observed for *Cx. pipiens* (Additional file 1: Table S1). A lower mean number of females of both species was collected in the outer side of the MET in the treated area compared to the untreated one (GLMM; p < 0.05, Additional file 1: Table S1).

Virtually no mosquito emergence was observed in the N-, S- and W-quarters of the treated area (Table 1, “inner face” and Table 3), while *Ae. albopictus* and *Cx. pipiens* emergence events in the two METs in the E-quarter (in the botanical garden) were comparable to those in the untreated area (E *vs* UN, Fisher exact test, p-value > 0.05; mean number of mosquito/trap in 48 hours (SE) = 4.1 (±0.6) and 8 (±0.8) *Ae. albopictus* and 3.6 (±0.7) and 5.8 (±0.7) *Cx. pipiens* in sites E and UN, respectively).

**Table 3:** ***Aedes albopictus*** **and** ***Culex pipiens*** **emergence events from larvicide-treated and untreated street catch basins**

	***Aedes albopictus***		***Culex pipiens***	
**Site**	**Females**	**Males**	**Females**	**Males**
Treated-N	0	0	0	1
Treated-E	11	15	11	14
Treated-S	0	0	0	2
Treated-W	0	0	0	1
Untreated	15	15	14	15

Adult female and male emergence events from two larvicide-treated catch basins in each of the four quadrants in the treated area and from two untreated catch basins during N = 15 (48-hour) sampling collections by Mosquito Emerging Trap.

### Evaluation of the efficacy of insecticide treatments on mosquito adult densities using Sticky Traps (ST) and Catch-Basin trap (CBT)

The ratio between *Ae. albopictus* and *Cx. pipiens* was higher in ST than in CBT in both areas (Table 2; Fisher’s Exact Test, p < 0.001 for all tests), as expected due to the high specificity of ST for *Ae. albopictus* [35].

**Figure 2:**
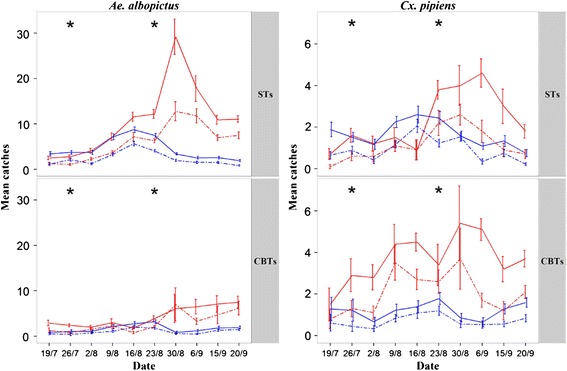
**Mosquito population dynamics in the insecticide treated area and in the untreated one.** Mean counts/trap/week of *Aedes albopictus* and *Culex pipiens* females (full lines) and males (dotted lines) collected by Sticky (ST) and Catch Basin (CBT) traps in insecticide-treated (blue line) and untreated (red line) areas. STs and CBTs were 36 in the treated area and 10 in the untreated one. X-axis: sampling dates in summer 2012; asterisks: dates of the two adulticide spraying in the treated area; vertical bars: standard errors.

The population dynamics of adult females and males of *Ae. albopictus* and *Cx. pipiens* in the two areas, as determined by ST and CBT collections, are summarized in Figure 2. The estimated percentage of control after the second application of adulticides (by Henderson’s method) was 80% and 87% for *Ae. albopictus* collections by ST and CBT, respectively, and 24% and 69% for *Cx. pipiens*.

**Figure 3:**
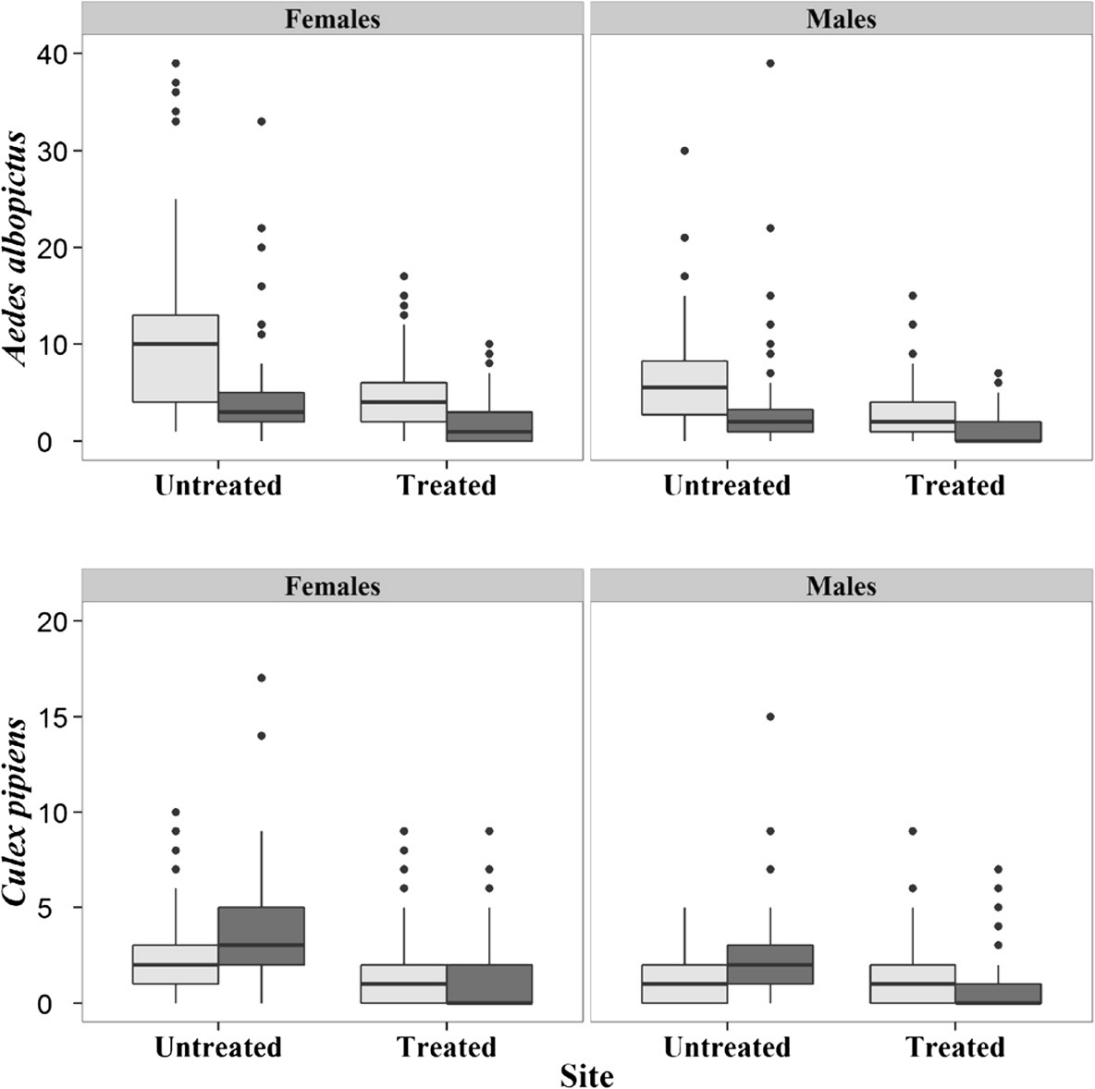
***Aedes albopictus*** **and** ***Culex pipiens*** **counts in the insecticide-treated and in the untreated areas.** Box-plots of adult female and male counts in 36 STs and 36 CBTs in the treated area and in 10 STs and 10 CBTs in the untreated one during the 10 weeks sampling in 2012. ST = Sticky Trap (light grey); CBT = Catch Basin trap (dark grey). The boxes identify the first and third quartiles (the 25th and 75th percentiles). The upper whisker extends from the boxes to the highest value that is within 1.5 * IQR (inter-quartile range: the distance between the first and third quartiles, so the height of the boxes). The lower whisker extends to the lowest value within 1.5 * IQR. Points beyond the end of the whiskers are outliers.

The results of the GLMM models performed to assess differences in overall abundance of mosquitoes between traps (ST *vs.* CBT) and between treated and untreated areas over the 10-week trapping period are given in Table 4 for females and Additional file 1: Table S2 for males: i) ST-counts were significantly higher than CBT-counts in each area for both *Ae. albopictus* females and males, corresponding to an overall difference of 58% and 48%, respectively (Figure 3); ii) CBT-counts were significantly higher than ST-counts in the untreated area for both *Cx. pipens* females and males, corresponding to an overall difference of 60% and 44.5% (Figure 3); iii) ST and CBT *Ae. albopictus* counts were significantly lower in the insecticide-treated than the untreated one (corresponding to a 60% and a 62% catch-difference for females and males, respectively) (Figure 2). iv) CBT *Cx. pipiens* counts were significantly lower in the insecticide-treated than in the untreated area (corresponding to a 68% and a 57% catch- difference for females and males, respectively) (Figure 2).

**Table 4:** **Results of generalized linear mixed model of female mosquito sampling in insecticide-treated** ***versus*** **untreated areas**

	***Aedes albopictus***				***Culex pipiens***			
**Parameter**	**Estimate**	**SE**	**z-value**	**Pr(>|z|)**	**Estimate**	**SE**	**z alue**	**Pr(>|z|)**
Intercept	2.33	0.12	18.46	<0.0001	0.82	0.12	7.09	<0.0001
Site (Treated)	−0.91	0.08	−10.74	<0.0001	−0.35	0.11	−3.06	0.0022
Trap (CBT)	−0.86	0.11	−7.99	<0.0001	0.47	0.13	3.54	0.0004
Site*Trap	−0.08	0.13	−0.62	0.53	−0.80	0.16	−4.99	<0.0001

Number of observation: 862, number of weeks: 10, SE: standard error of parameter estimate, z-value: estimate to standard error ratio, Pr(>|z|): statistic for z-value. Untreated area and Sticky Trap as reference level (CBT = Catch Basin Traps).

However, no statistical differences in *Ae. albopictus* and *Cx. pipiens* females and males counts between treated and untreated area were observed when considering collections carried out in the 2 weeks before the first adulticide treatment (Additional file 1: Tables S3 and S4), nor those carried out in the 6 weeks before the second one (Additional file 1: Tables S5 and S6). In all GLMMs no spatial autocorrelation was observed (i.e. the variogram did not show any clear violation of independence).

A positive relationship was observed between ST- and CBT-counts both for *Ae. albopictus* (GLM output: Chi-square = 20.85, df = 1, P < 0.001) and for *Cx. pipiens* (Chi-square = 29.86, df = 1, P < 0.001) females within the treated area only.

**Figure 4:**
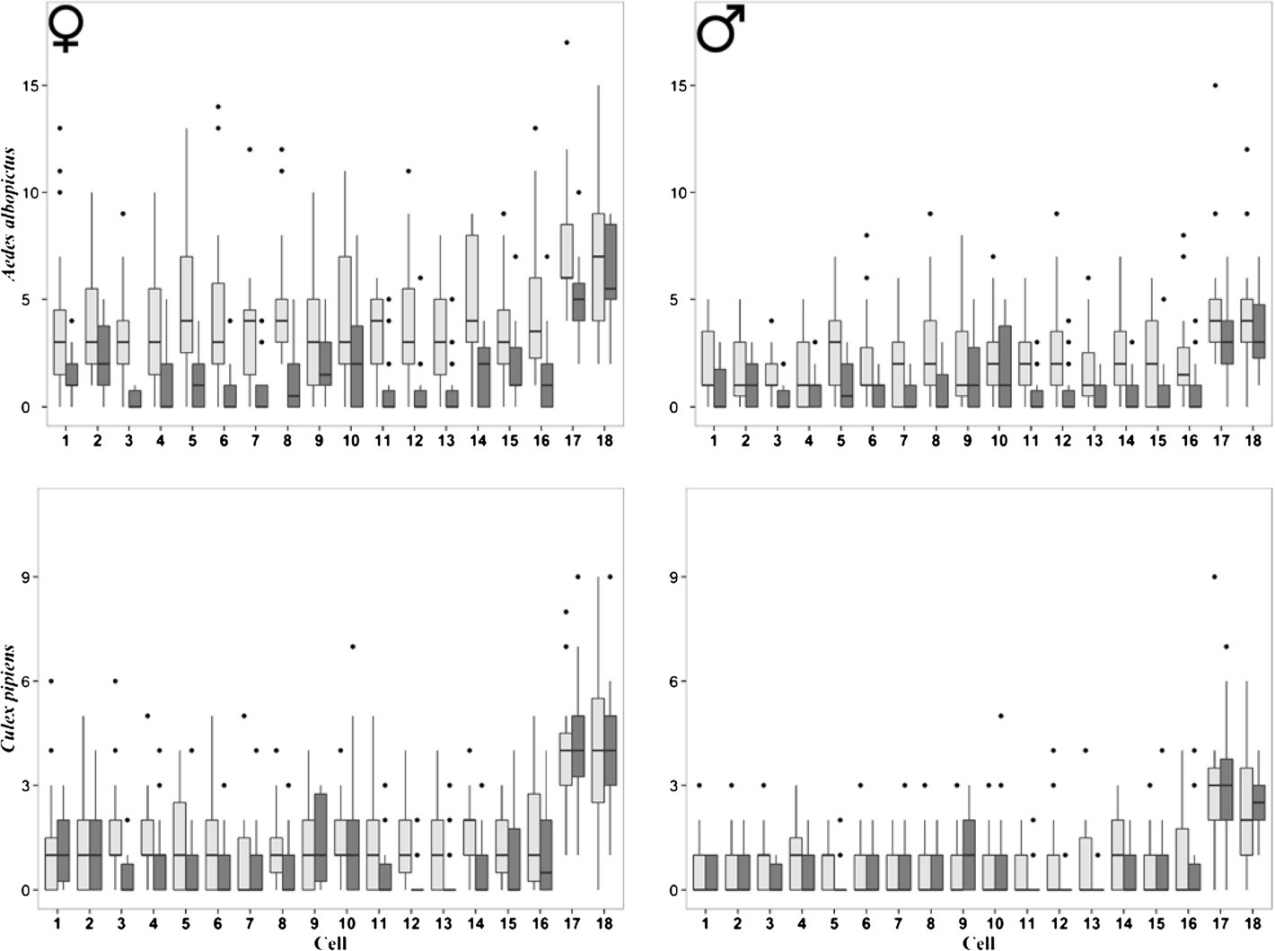
***Aedes albopictus*** **and** ***Culex pipiens*** **counts in each of the 18 cells of the insecticide-treated area.** Box-plots of adult female and male counts in 2 Sticky-Traps (STs; light grey) and 2 Catch Basin Traps (CBTs; dark grey) in each of the 18 cells of the insecticide-treated area during the 10-week sampling in 2012. The boxes identify the first and third quartiles (the 25th and 75th percentiles). The upper whisker extends from the boxes to the highest value that is within 1.5 * IQR (inter-quartile range: the distance between the first and third quartiles, so the height of the boxes). The lower whisker extends to the lowest value within 1.5 * IQR. Points beyond the end of the whiskers are outliers.

Finally, the results of the GLMMs carried out to assess possible spatial differences in mosquitoes counts among the 18 cells within the treated area in the entire 10-week sampling period (Figure 4; Table 5; Additional file 1: Table S7) showed: i) significantly higher mosquito counts in cell-17 and −18; ii) significantly higher *Ae. albopictus* counts in ST than in CBT in all cells, except cell-18; iii) no difference in *Cx. pipiens* counts between the two traps in all the cells. Again, in all GLMMs no spatial autocorrelation was observed.

**Table 5:** **Results of generalized linear mixed model of female mosquito sampling in 18-cells within the insecticide-treated area**

	***Aedes albopictus***				***Culex pipiens***			
**Parameter**	**Estimate**	**SE**	**z value**	**Pr(>|z|)**	**Estimate**	**SE**	**z value**	**Pr(>|z|)**
Intercept	1.26	0.18	6.93	<0.0001	0.27	0.22	1.25	0.21
Trap (CBT)	−0.80	0.22	−3.64	0.0002	−0.11	0.30	−0.37	0.71
Cell 17	0.67	0.16	4.29	<0.0001	1.04	0.24	4.30	<0.0001
Cell 18	0.62	0.16	3.92	<0.0001	1.10	0.24	4.60	<0.0001
CBT* Cell 17	0.38	0.27	1.43	0.15	0.22	0.35	0.62	0.54
CBT* Cell 18	0.62	0.27	2.34	0.02	0.10	0.35	0.30	0.76

Number of observation: 662, number of weeks: 10, SE: standard error of parameter estimate, z-value: estimate to standard error ratio, Pr(>|z|): statistic for z-value. Cell 1 and Sticky Trap as reference level. (CBT = Catch Basin Traps).

## Discussion

The present results support the exploitation of adhesive traps to monitor adult mosquitoes in an urban environment and to assess the efficacy of insecticide treatments against them*.* This latter aspect is particularly relevant, as the efficacy of insecticide control activities is rarely, if ever, evaluated in European urban areas where these activities are carried out to reduce mosquito nuisance rather than to reduce the risk of pathogen transmission. This is due to a lack of standardized and simple devices to collect adults urban mosquitoes and of standardized easy-to-use procedures to take into account all possible interacting variables (e.g. climatic factors, insecticide characteristics, spraying methods, etc.), as well as of appropriate motivation and resources.

The newly designed MET allows to discriminate between mosquitoes freshly emerged from catch basins and those visiting the basins either for laying eggs or for resting. Thus MET could be exploited to assess the percentage of positive catch basins, thus providing an index similar to those commonly utilized to evaluate the positivity of other types of water containers (e.g. Container Index = percentage of inspected water-holding containers infested with larvae or pupae). MET has the advantage to directly assess the presence of emerging adults (as opposed to larvae or pupae) without the need of high numbers of larval dips inside the catch basins (which are needed to collect a representative number of larvae/pupae [47]), thus also eliminating the need of rearing larvae to the adult stage, when species with similar larval morphology are present [48]. Moreover, MET can be exploited to assess the lethal effect of larvicide-treatments in catch basins, which represent a fundamental component in the control of the abundance of urban mosquitoes [49] and are very commonly carried out in infested municipalities in Italy [21]. Results obtained showed that, although all monitored catch basins were shown to be visited by *Ae. albopictus* and *Cx. pipiens*, adult emergence was completely inhibited in most diflubenzuron-treated basins (the few exceptions are discussed below). It should be stressed that results obtained by this approach do not allow an assessment of mortality rates, which requires collection of larvae/pupae in treated catch basins followed by laboratory observations, particularly needed when IGR-analogs, which acts not only on larvae but also on pupae, are used. MET can thus be proposed to more easily assess directly in the field whether the treatments are actually achieving the expected goal, i.e. the “sterilization” of the catch basins. Moreover, MET could simultaneously allow an evaluation of the effect of larvicide on adults abundance based on mosquito collected on its outer side. Possible limitations for MET exploitation may be represented by heavy rains and presence of abundant debris which could reduce the adhesive properties of the trap and the morphological qualities of collected specimens. Under our climatic conditions and with the experimental protocol applied neither limitations represented a major problem in the present study.

In contrast to MET, CBT does not allow to discriminate between freshly emerged specimens and those visiting the catch basins for resting/ovipositing, and thus is not a good tool to assess efficacy of larvicide treatments in the catch basin. However, since CBT is easily installed perpendicular to the drain-grid (and does not require the lift of the grid needed to locate MET), CBT can be exploited for large scale monitoring of urban mosquitoes associated with catch basins. Results from the untreated area indicated that CBT collected higher numbers of *Cx. pipiens* and lower numbers of *Ae. albopictus* when compared to ST. This suggests that CBT has the potential to be successfully exploited to passively monitor *Cx. pipiens* abundance and dynamics in urban areas. In fact, monitoring of this species - which represents a major vector of zoonotic pathogens such as West Nile Virus [50] and *Dirofilaria* worms [51] - almost exclusively relies on traps requiring a fan driven by an electric motor and more complicate logistics (e.g. CDC-light traps, CDC gravid trap). On the other hand, ST is confirmed to be an effective device for monitoring *Ae. albopictus* [35]. In fact, although it only targets ovipositing and resting adults, it collected more individuals than CBT, which also targets freshly emerged adults. This suggests that in the urban environment *Ae. albopictus* is less attracted to catch basins than to smaller oviposition/resting sites, which more closely resemble its original sylvatic larval habitats (e.g. tree-holes, rock-holes and fruit husks [8]).

Results also showed that ST and CBT are effective tools for assessing abundance and population dynamics of adult *Ae. albopictus* (and *Cx. pipiens*) in an area where catch basins were treated by IGR-analogs and two insecticide Low Volume sprayings were carried out before sunsets. Both ST and CBT have major advantages compared to other tools usually used to evaluate the effect of control activities against *Ae. albopictus*, e.g. BG-sentinel traps or ovitraps [27,34], both of which have major conceptual and operational constraints. BG-sentinel traps are expensive, require power supply and release of CO_2_ and synthetic lures to attract and collect mosquito adults. Ovitraps provide only indirect estimates of adult abundance based on numbers of eggs collected. In addition, in regions where species with similar egg/larval morphology (e.g. *Aedes aegypti, Ochlerotatus geniculatus*) coexist, larvae must be reared to late instar or adults for reliable identification. On the other hand, ST and CBT are cheap, easy to manage (and can thus be deployed in larger numbers) and allow easy identification of gender and species. During the testing in Rome, we found ST to be much more efficient than CBT for monitoring the impact of control measures on *Ae. albopictus*, while the opposite was true for *Cx. pipiens.* Moreover, the concomitant use of ST and CBT provided result in agreement with the lethal effect of diflubenzuron treatments in catch basins (already revealed by MET, see above). In fact, GLM analysis demonstrated a direct relationship between *Ae. albopictus* and *Cx. pipiens* counts by ST and CBT in the treated area, but not in the untreated one. The lack of a direct relationship between CBT- and ST-counts in the untreated area is likely due to the fact that ST only collects mosquitoes visiting the catch basin, either for resting or ovipositing, whereas CBT also collects freshly emerged adults. On the other hand, the direct relationship between CBT- and ST-counts in the treated area is likely due to lack of emergent adults in an area were catch basins are regularly treated with IGR-analogs.

The abundance of *Ae. albopictus* and *Cx. pipiens* was shown to be significantly lower in the treated vs untreated area only after the second adulticide spraying. This may be due to our sampling effort which could not have had a high enough resolution to detect the impact of the first treatment. In fact, this treatment was carried out in late July when adult densities of *Ae. albopictus* were low, whereas the second was carried out four weeks later when the *Ae. albopictus* population was growing [52]. However, it is possible that the results obtained highlighted an actual different impact of the two treatments on the mosquito adult population. Interestingly, the mosquito population expansion was evident in the untreated site in August but was completely absent in the treated site for both females and males (Figure 2). Noteworthy, the estimated percentage of control observed after the second spraying (i.e. 80% and 87%, as estimated by ST and CBT collections, respectively) is similar to that obtained after a single night-time ultra-low volume (ULV) treatment with DUET™ Dual-action Adulticide (Clarke H, Roselle, IL, USA) in New Jersey (USA) (i.e. 73% as estimated by BG-trap collections; [34]). To our knowledge, our results represent the first preliminary indication of the effectiveness of LV spraying against *Ae. albopictus* in urban areas, which is relevant, as ULV spraying is extensively applied in the US and some European Countries [53], but not in Italy. The high impact observed in Rome may have been due to the timing (before sunset rather than at night as in New Jersey) which corresponds to the peak of flight activity of *Ae. albopictus*), when the treatments are supposed to be most effective [54]. Moreover, the effect of treatment may have been enhanced and protracted by the fact that the study area, although limited in size, is surrounded by a ∼ 3 m-high wall which, based on the relatively low flight height of *Aedes albopictus* [8], may have acted as a barrier to re-introduction of large numbers of adults from neighbouring areas, thus maximizing the effect of the spraying.

The accurate monitoring of the treated area with high numbers of STs and CBTs revealed spatial heterogeneities in the treatment efficacy, which was shown to be lower in the botanical garden than in the rest of the treated area (Figure 1a). This may be due to several factors. First, insecticide spraying was restricted to the perimeter of the garden where the dense vegetation may have blocked movement of the insecticide aerosol (as reported by [54]). Moreover, the regular and abundant watering of the plants may have diluted larvicide concentration in the catch basins. In fact, MET revealed that the only adult emergence was in the two catch basins in the botanical garden, where it was comparable to that in the untreated sites. These results indicate the need to identify hot-spots of mosquito production (e.g. small public or private gardens)—as well as of resting sites—in order to maximize the impact of mosquito control activities and to achieve an overall successful reduction of mosquito abundance and nuisance.

## Conclusions

The results support the potential of the three adhesive traps tested in passively monitoring urban mosquito adult abundance and seasonal dynamics and in assessing the efficacy of control measures. The results also provide a preliminary indication on the effectiveness of IGR-treatments in catch basins carried out during the whole *Ae. albopictus* reproductive season in association with LV insecticide spraying carried out during the beginning of the major population expansion in reducing adult abundance. It is important to remind that in this study adulticide treatments were carried out before sunset, when they are expected to be most effective, while in most urban areas for safety reasons the treatments can be carried out only during night-time or very early in the morning [20,21]. More data are needed to: a) confirm the impact of adulticide treatments carried out i) during the night, ii) at different phases of the mosquito seasonal dynamics; iii) under different ecological conditions, and b) to assess the relative role of larvicide and adulticide treatments. Anyhow, the present results stress the potential benefits of adulticide treatments consequent to an accurate monitoring of adult densities. The latter would also have the benefit to identify hot-spots of larval/resting sites, which need to be specifically targeted in order to maximize the impact of the control campaign. It is however important to stress that insecticide spraying should represent a method reserved for emergency response and that a preferred strategy to control *Ae. albopictus* should include other integrated activities [26] - such as larval source reduction [55,56], biological control [57], education and public awareness, as well as personal protection [58]– which are often neglected by the competent authorities.

Finally, it would also be interesting to correlate collections by ST and/or CBT with levels of mosquitoes nuisance to establish a “threshold of nuisance” (as it has been done for ovitraps [59], and BG-sentinel traps [27]) over which adulticide treatments could be recommended not only to reduce the burden of this aggressive mosquito biter to the citizens, but also to prevent the establishment of densities which would represent a high risk in the case of migrant humans infected by arboviruses, as it occurred in 2007 with Chikungunya virus in Italy [9].

## Additional file

Additional file 1: Table S1. P-values of Generalized Linear Mixed Model analysis of mosquitoes visiting METs in larvicide-treated and untreated areas. **Table S2.** Results of Generalized Linear Mixed Model of male mosquito sampling in insecticide-treated *versus* untreated areas. **Table S3.** Results of Generalized Linear Mixed Model analysis of female mosquito sampling in insecticide-treated *versus* untreated areas before the first insecticide-spraying. **Table S4.** Results of Generalized Linear Mixed Model analysis of male mosquito sampling in insecticide-treated *versus* untreated areas before the first insecticide-spraying. **Table S5.** Results of Generalized Linear Mixed Model analysis of female mosquito sampling in insecticide-treated *versus* untreated areas before the second insecticide-spraying. **Table S6.** Results of Generalized Linear Mixed Model analysis of male mosquito sampling in insecticide-treated *versus* untreated areas before the second insecticide-spraying. **Table S7.** Results of Generalized Linear Mixed Model of male mosquito sampling in 18-cells within the insecticide-treated area.
