# Supplementary material for: New adhesive traps to monitor urban mosquitoes with a case study to assess the efficacy of insecticide control strategies in temperate areas
Source: Parasit Vectors. 2015 Feb 28;8:134. doi: 10.1186/s13071-015-0734-4 (PMC4378381; doi:10.1186/s13071-015-0734-4)
Supplement: Additional file 1: Table S1. — P-values of Generalized Linear Mixed Model analysis of mosquitoes visiting METs in larvicide-treated and untreated areas. Table S2. Results of Generalized Linear Mixed Model of male mosquito sampling in insecticide-treated versus untreated areas. Table S3. Results of Generalized Linear Mixed Model analysis of female mosquito sampling in insecticide-treated versus untreated areas before the first insecticide-spraying. Table S4. Results of Generalized Linear Mixed Model analysis of male mosquito sampling in insecticide-treated versus untreated areas before the first insecticide-spraying. Table S5. Results of Generalized Linear Mixed Model analysis of female mosquito sampling in insecticide-treated versus untreated areas before the second insecticide-spraying. Table S6. Results of Generalized Linear Mixed Model analysis of male mosquito sampling in insecticide-treated versus untreated areas before the second insecticide-spraying. Table S7. Results of Generalized Linear Mixed Model of male mosquito sampling in 18-cells within the insecticide-treated area. [file 13071_2015_734_MOESM1_ESM.doc]

**SUPPLEMENTARY MATERIALS**

**Table S1 – P-values of Generalized Linear Mixed Model analysis of mosquitoes visiting METs in larvicide-treated and untreated areas.**

Results from comparison of mosquito collections in the outer side of Mosquito Emerging Trap (MET) among METs located in each quarter of the insecticide-treated area (N, E, S, W) and in the untreated area (UN) during the N=15 (48-hour) samplings in 2012.

| ***Aedes albopictus* females** | | | | | ***Culex pipiens* females** | | | |
| --- | --- | --- | --- | --- | --- | --- | --- | --- |
|  | **N** | **E** | **W** | **S** | **N** | **E** | **W** | **S** |
| **UN** | <0.001 | 0.02 | 0.003 | <0.001 | <0.001 | 0.021 | 0.001 | <0.001 |
| **N** |  | 0.002 | 0.015 | 0.896 |  | 0.119 | 0.64 | 0.752 |
| **E** |  |  | 0.5 | 0.003 |  |  | 0.27 | 0.210 |
| **W** |  |  |  | 0.021 |  |  |  | 0.879 |

**Table S2** – **Results of Generalized Linear Mixed Model of male mosquito sampling in insecticide-treated *versus* untreated areas.**

Number of observation=862, Number of weeks =10, SE: standard error of parameter estimate, z-value: estimate to standard error ratio, Pr(>|z|): statistic for z-value. Untreated area and ST trap as reference level.

|  | ***Aedes albopictus*** | | | | ***Culex pipiens*** | | | |
| --- | --- | --- | --- | --- | --- | --- | --- | --- |
| Parameter | Estimate | SE | z value | Pr(>|z|) | Estimate | SE | z value | Pr(>|z|) |
| Intercept | 1.72 | 0.15 | 11.03 | <0.0001 | 0.06 | 0.18 | 0.34 | 0.73 |
| Site (Treated) | -0.97 | 0.09 | -9.97 | <0.0001 | -0.24 | 0.14 | -1.72 | 0.08 |
| Trap (CBT) | -0.65 | 0.12 | -5.41 | <0.0001 | 0.59 | 0.16 | 3.70 | 0.0002 |
| Site*Trap | -0.12 | 0.14 | -0.83 | 0.41 | -0.84 | 0.19 | -4.40 | <0.0001 |

**Table S3** – **Results of Generalized Linear Mixed Model analysis of female mosquito sampling in insecticide-treated *versus* untreated areas before the first insecticide-spraying**.

Number of observation: 128, Number of weeks: 2, SE: standard error of parameter estimate, z-value: estimate to standard error ratio, Pr(>|z|): statistic for z-value. Untreated area and ST trap as reference level.

|  | ***Aedes albopictus*** | | | | ***Culex pipiens*** | | | |
| --- | --- | --- | --- | --- | --- | --- | --- | --- |
| Parameter | Estimate | SE | z value | Pr(>|z|) | Estimate | SE | z value | Pr(>|z|) |
| Intercept | 0.97 | 0.15 | 6.36 | <0.0001 | 0.14 | 0.29 | 0.48 | 0.63 |
| Site (Treated) | 0.32 | 0.18 | 1.81 | 0.07 | 0.35 | 0.33 | 1.05 | 0.29 |
| Trap (CBT) | 0.00 | 0.22 | 0.00 | 1 | 0.65 | 0.38 | 1.68 | 0.093 |
| Site*Trap | -1.35 | 0.29 | -4.60 | <0.0001 | -0.92 | 0.47 | -1.95 | 0.051 |

**Table S4** - **Results of Generalized Linear Mixed Model analysis of male mosquito sampling in insecticide-treated *versus* untreated areas before the first insecticide-spraying**.

Number of observation=128, Number of weeks =2, SE: standard error of parameter estimate, z-value: estimate to standard error ratio, Pr(>|z|): statistic for z-value . Untreated area and ST trap as reference level.

|  | ***Aedes albopictus*** | | | | ***Culex pipiens*** | | | |
| --- | --- | --- | --- | --- | --- | --- | --- | --- |
| Parameter | Estimate | SE | z value | Pr(>|z|) | Estimate | SE | z value | Pr(>|z|) |
| Intercept | 0.18 | 0.24 | 0.77 | 0.44 | -1.05 | 0.44 | -2.36 | 0.018 |
| Site (Treated) | 0.42 | 0.27 | 1.56 | 0.11 | 0.84 | 0.49 | 1.70 | 0.09 |
| Trap (CBT) | -0.13 | 0.34 | -0.39 | 0.70 | 1.10 | 0.55 | 2.00 | 0.045 |
| Site*Trap | -1.16 | 0.44 | -2.61 | 0.001 | -1.52 | 0.65 | -2.33 | 0.02 |

**Table S5** - **Results of Generalized Linear Mixed Model analysis of female mosquito sampling in insecticide-treated *versus* untreated areas before the second insecticide-spraying**.

Number of observation: 495, Number of weeks: 6, SE: standard error of parameter estimate, z-value: estimate to standard error ratio, Pr(>|z|): statistic for z-value. Untreated area and ST trap as reference level.

|  | ***Aedes albopictus*** | | | | ***Culex pipiens*** | | | |
| --- | --- | --- | --- | --- | --- | --- | --- | --- |
| Parameter | Estimate | SE | z value | Pr(>|z|) | Estimate | SE | z value | Pr(>|z|) |
| Intercept | 1.79 | 0.18 | 9.86 | <0.0001 | 0.44 | 0.17 | 2.53 | 0.011 |
| Site (Treated) | -0.12 | 0.08 | -1.46 | 0.144 | 0.20 | 0.16 | 1.29 | 0.198 |
| Trap (CBT) | -0.85 | 0.12 | -7.02 | <0.0001 | 0.72 | 0.18 | 3.91 | <0.0001 |
| Site*Trap | -0.27 | 0.14 | -1.87 | 0.06 | -1.18 | 0.22 | -5.47 | <0.0001 |

**Table S6** - **Results of Generalized Linear Mixed Model analysis of male mosquito sampling in insecticide-treated *versus* untreated areas before the second insecticide-spraying**.

Number of observation=495, Number of weeks =6, SE: standard error of parameter estimate, z-value: estimate to standard error ratio, Pr(>|z|): statistic for z-value. Untreated area and ST trap as reference level.

|  | ***Aedes albopictus*** | | | | ***Culex pipiens*** | | | |
| --- | --- | --- | --- | --- | --- | --- | --- | --- |
| Parameter | Estimate | SE | z value | Pr(>|z|) | Estimate | SE | z value | Pr(>|z|) |
| Intercept | 1.15 | 0.22 | 5.14 | <0.0001 | -0.198 | 0.25 | -0.79 | 0.43 |
| Site (Treated) | -0.19 | 0.09 | -2.04 | 0.041 | 0.15 | 0.19 | 0.83 | 0.41 |
| Trap (CBT) | -0.85 | 0.14 | -5.92 | <0.0001 | 0.79 | 0.21 | 3.74 | 0.0002 |
| Site*Trap | -0.11 | 0.17 | -0.65 | 0.516 | -1.14 | 0.25 | -4.57 | <0.0001 |

**Table S7 - Results of Generalized Linear Mixed Model of male mosquito sampling in 18-cells within the insecticide-treated area**

Number of observation: 662, number of weeks: 10, SE: standard error of parameter estimate, z-value: estimate to standard error ratio, Pr(>|z|): statistic for z-value. Cell 1 and Sticky Trap as reference level. (CBT= Catch Basin Traps).

|  | ***Aedes albopictus*** | | | | ***Culex pipiens*** | | | |
| --- | --- | --- | --- | --- | --- | --- | --- | --- |
| Parameter | Estimate | SE | z value | Pr(>|z|) | Estimate | SE | z value | Pr(>|z|) |
| Intercept | 0.50 | 0.22 | 2.24 | 0.0252 | -0.74 | 0.35 | -2.14 | 0.032 |
| Trap (CBT) | -0.83 | 0.31 | -2.70 | 0.0069 | -0.46 | 0.52 | -0.88 | 0.377 |
| Cell 17 | 0.91 | 0.20 | 4.57 | <0.0001 | 1.67 | 0.35 | 4.75 | <0.0001 |
| Cell 18 | 0.85 | 0.20 | 4.24 | <0.0001 | 1.46 | 0.36 | 4.08 | <0.0001 |
| CBT* Cell 17 | 0.46 | 0.35 | 1.32 | 0.1861 | 0.47 | 0.56 | 0.84 | 0.401 |
| CBT* Cell 18 | 0.59 | 0.35 | 1.68 | 0.0935 | 0.55 | 0.57 | 0.97 | 0.335 |
